# Prevalence of different types of interproximal contacts in the permanent dentition – a study cast evaluation

**DOI:** 10.12688/wellcomeopenres.18973.2

**Published:** 2023-10-24

**Authors:** Vignesh Kailasam, MS Muthu, Usha Rao, Krithika C, M Kirthiga, J Aarthi, Sankara Aravind Warrier

**Affiliations:** 1 Department of Orthodontics and Dentofacial Orthopedics, Sri Ramachandra Dental College and Hospital, Sri Ramachandra Institute of Higher Education and Research, Chennai, India; 2 Centre for Early Childhood Caries Research (CECCRe), Department of Pediatric and Preventive Dentistry, Sri Ramachandra Dental College and Hospital, Sri Ramachandra Institute of Higher Education and Research, Porur, Chennai, India; 3 Adjunct Research Associate, Centre of Medical and Bio-Allied Health Sciences Research, Ajman University, Ajman, United Arab Emirates; 4 Professor, Department of Orthodontics and Dentofacial Orthopedics, Tamil Nadu Government Dental College, Chennai, Tamil Nadu, India; 5 Registrar, Professor, Oral Medicine and Radiology, Meenakshi Academy of Higher Education and Research, Chennai, India; 6 Reader, Department of Paediatric and Preventive Dentistry, Madha Dental College, Chennai, India; 7 Professor and Head, Department of Oral Medicine and Radiology, Sri Ramachandra Dental College and Hospital, Sri Ramachandra Institute of Higher Education and Research, Chennai, India

**Keywords:** Andrews fifth key, Interproximal contact, Occlusion, OXIS classification, Permanent Dentition.

## Abstract

**Background:**

A new classification called OXIS was proposed for categorizing the interproximal contacts of primary molars, modified for the primary canines and its prevalence was established. No such information is available for the permanent dentition. Hence, the aim was to establish the variations in interproximal contacts of the permanent dentition and thereby modify the OXIS classification of primary molars and primary canines to the permanent dentition.

**Methods:**

We propose a study-cast-based classification of interproximal contacts of the permanent dentition. Three hundred and forty-three pretreatment casts of patients based on an inclusion and exclusion criteria were selected. Contacts of posterior teeth were classified based on OXIS classification of interproximal contacts, and its modification was used for anterior teeth.

**Results:**

Among the posterior contacts, the ‘O’ type of contact was least prevalent, while most prevalent was the ‘S’ type for second molar-first molar contact, ‘I’ type for the first molar-second premolar contact, and ‘X’ type for the second premolar-first premolar contact. Among the anterior contacts, least prevalent was ‘S1’ type for the first premolar-canine contact, and I type for the canine-lateral incisor and the lateral incisor-central incisor contacts. There was no statistical significance between right- and left-side contacts ( *P* > 0.05) while significance was seen between maxillary and mandibular contacts ( *P* < 0.05). Similarity of contacts ranged from 5.17% to 10.05%.

**Conclusion:**

The OXIS classification is applicable to posterior permanent teeth, and its modification is representative of anterior permanent teeth.

## Introduction

The six keys to normal occlusion, namely, molar relationship, crown angulation, crown inclination, rotations, tight contacts, and occlusal plane, were proposed by Andrews ^1^. The fifth key, tight contacts, refers primarily to the nature of the interproximal contacts. An intact and a well-founded inter proximal contact plays a major role in the periodontal health and stability of the dentition ^2^.

Contact areas have been studied and classified in the primary molars as being of four different types, namely, open (O), point contact (X), straight contact (I), and a curved contact (S), and thus the acronym “OXIS” ^2^. For the interproximal contacts of the primary canines, a modification of the OXIS classification has recently been reported ^3^. While the OXIS classification is of particular significance to the occurrence of dental caries, it would impact class II cavity preparation, placement of stainless steel crowns as well as interproximal reduction (IPR). Existing literature suggests primary teeth which are characterized by broader inter proximal contact areas have a greater susceptibility to caries ^4– 6^. Further, Cortes *et al.* have reported that in primary molar teeth, if both the proximal surfaces are concave, the caries risk is more ^7^.

While contact areas have been studied and classified in the primary dentition, no such information is available for the permanent dentition except that it is listed as the fifth of the six keys to occlusion. The variations in the contact areas of the permanent dentition might alter caries susceptibility and have orthodontic implications affecting the magnitude of force application, force distribution from one tooth to another, and stability of orthodontic treatment. Also, extraction decisions in orthodontics have been based on post-treatment contacts. First premolars are routinely extracted to maintain first-molar–second-premolar continuity rather than placing the molar adjacent to the first premolar, which is not as anatomically suitable for molar contact ^8^. Further research into the types of and variations in interproximal contact areas will elucidate the validity of this decision. Thus, a classification of the interproximal contacts in the permanent dentition would be the first step to providing more information on the orthodontic implications and importance of the fifth key of occlusion. A classification based on study casts would not only ensure simplicity but could also be easily applied during a routine intraoral clinical examination. Hence, the aim was to establish the variations in interproximal contacts of the permanent dentition and thereby modify the OXIS classification of primary molars and primary canines to the permanent dentition and report the prevalence of the same.

## Methods

### Study design and ethics approval

Institutional Ethics Committee, (REF: IEC-NI/19/JUL/70/50) approval was obtained for this cross-sectional retrospective study. Casts of the patients were taken from department records. Written Informed consent was obtained from the patients.

***Training and calibration of the examiner*.** Before the study began in the university orthodontic department, a single dentist VK was extensively trained and calibrated under the supervision of one of the authors of the OXIS classification, to evaluate the contact areas. The calibration process involved a presentation of the classification and theoretical discussions on its use, followed by practical sessions thereon. Finally, a clinical exercise involving examination and re-examination of the same 20 patient study casts (26 intact contacts per patient), comprising 520 intact contacts, was screened by the same primary investigator on two separate occasions after a three-week interlude. The kappa value to test the intra-examiner variability was obtained as 0.82, which reflected a high degree of agreement.

***Sample size calculation*.** Sample size calculation was based on the data from a pilot study of 780 intact interproximal contacts (30 patient casts) The distribution of the types of contacts in percentage terms was as follows: O type, 16%; X type, 13%; I type, 62%; and S type, 9%. Therefore, with an expected proportion of 9%, relative precision of 20%, and desired confidence level of 95%, the sample size required was 8917 contacts, that is, 343 patient casts. 

***Inclusion and exclusion criteria*.** The inclusion criteria were: casts of patients with intact contacts, with a full complement of permanent teeth other than third molars, and between the ages of 14 years and 25 years. The exclusion criteria were casts of patients with carious, filled or fractured teeth, occlusal wear and any developmental anomalies affecting the morphology of the tooth (peg laterals, etc.) After an initial screening of 622 patient casts obtained from the records of the Department of Orthodontics (XXX), 343 patient casts that matched the eligibility criteria were selected. The records obtained were from the period 2016 to 2020.

**Figure 1.**
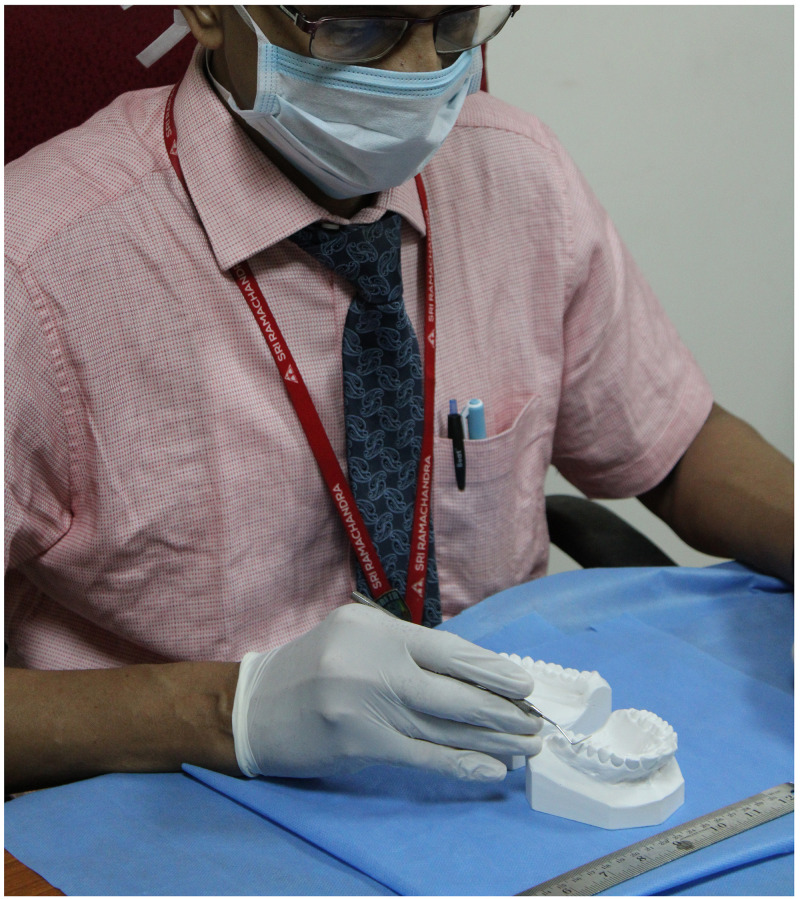
Image depicting standardization of distance during interproximal contact assessment.

***Assessment and evaluation*.** The examiner classified not more than 25 patient casts a day in random order. The evaluation was done with the naked eye and natural lighting. These casts were one-to-one replicas without magnification. They were based and placed on a flat table for assessment, and the evaluation was done from a standardized distance. The distance was standardized by placing the casts on a specified table. The distance between the eye line of the primary investigator and the cast was measured to be one foot. These standardizations were applied for all measurements ( Figure 1). The assessment of the maxillary and mandibular casts always began with the second molar-first molar interproximal contact on the right quadrant and proceeded across the midline to the left quadrant second molar-first molar interproximal contact. Whenever there was any doubt regarding the quantum of measurement with visual inspection, a Williams probe was used to measure the distance and the contact was graded accordingly. The shape of the evaluated contact was simultaneously entered into the data sheet designed for this study. Third molars were not considered.

***Evaluation of posterior tooth contacts*.** The maxillary and mandibular posterior teeth were examined by being viewed from the occlusal surface for the shapes of the contact areas. The posterior tooth contacts assessed were three per quadrant, that is, the contact between the mesial aspect of the second molar and the distal aspect of the first molar, up to the contact between the mesial aspect of the second premolar and the distal aspect of the first premolar. This was done for right and left sides and for the maxillary and mandibular casts. The contacts were classified based on the OXIS classification ^2^. Briefly, this classification applied ’O‘, that is, an open contact, if there was no contact between the adjacent teeth, ’X’ if there was a point of contact of less than or equal to 1.5 mm, ’I’ if there was a straight contact of more than 1.5 mm, and ’S’ if there was a curved contact of greater than 1.5 mm between adjacent teeth.

***Evaluation of anterior tooth contacts*.** The maxillary and mandibular anterior teeth were examined by being viewed from the incisal surface for the shapes of the contact areas. Contacts between mesial aspect of first premolar and distal aspect of canine, starting from right side to the corresponding tooth contact on the left side, were assessed. The anterior tooth contacts were classified based on a modification of the OXIS classification ^3^, where the ‘S’ type alone was modified as ‘S1’ or ‘S2’. The contact was classified as ‘S’ type I (S1) when the tooth was rotated and only one of its surfaces (either proximal or labial/lingual) was in contact with the adjacent tooth. The contact was classified as ‘S’ type II (S2) when the tooth was rotated and had two surfaces — proximal (mesial/distal) and labial or lingual was in contact with the adjacent tooth.

**Figure 2.**
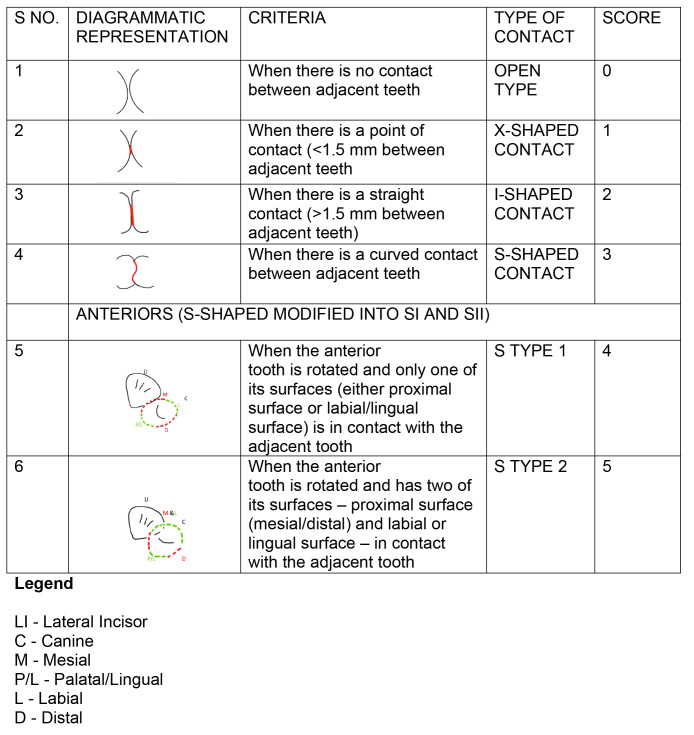
Pictorial representation of the OXIS classification.

***Scoring*.** For posterior contacts, a score of 0 was given for the open (O) contacts 1 for ‘X’, 2 for ‘I’ and 3 for ‘S’. For anterior contacts, the ‘O’, ‘X’, and ‘I’ contacts were also scored as 0, 1, and 2, but the ‘S’ type ‘I’ was scored as 3, and the ‘S’ type II was scored as 4. Figure 2– Figure 6 are illustrations of the OXIS patterns of the interproximal contacts, with their corresponding clinical images and their equivalent stone models.

**Figure 3.**
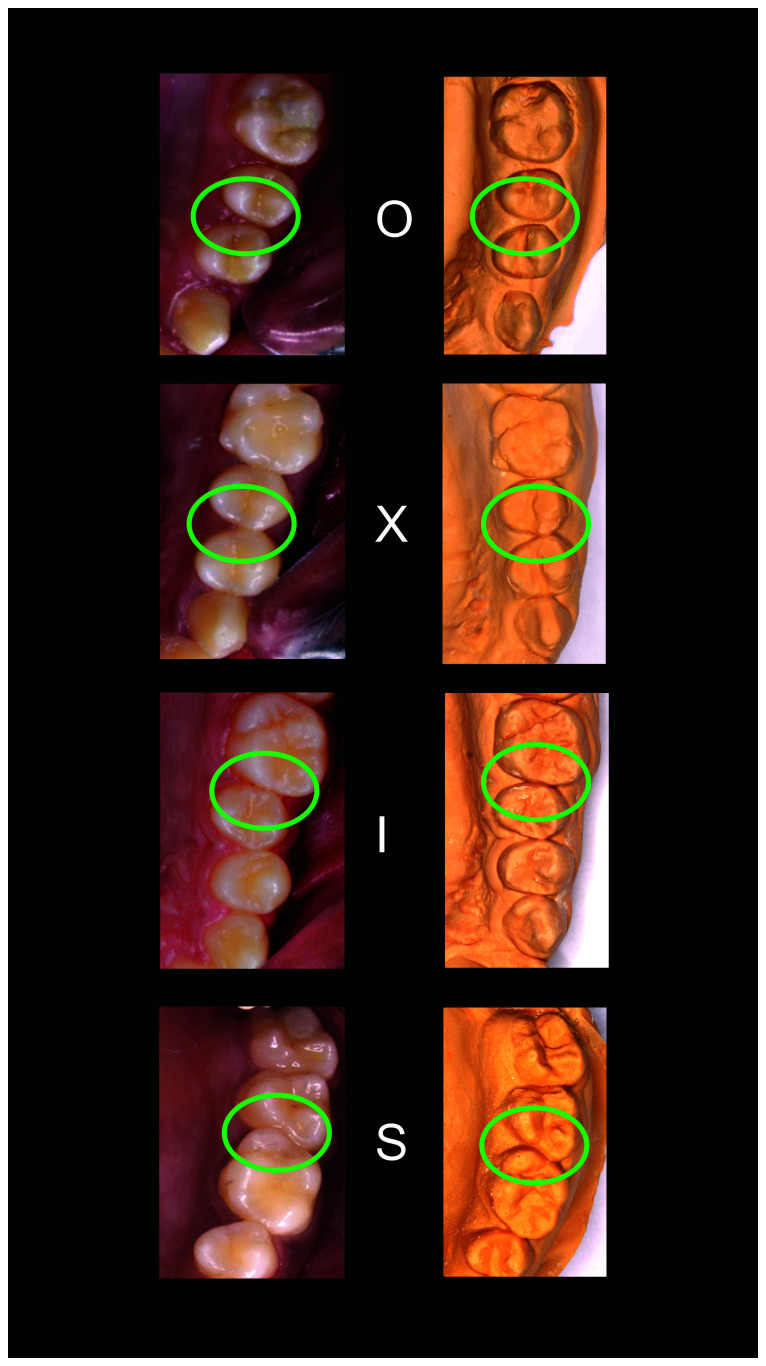
Representative cast images and their equivalent clinical photographs of the interproximal contacts of the maxillary posterior region.

**Figure 4.**
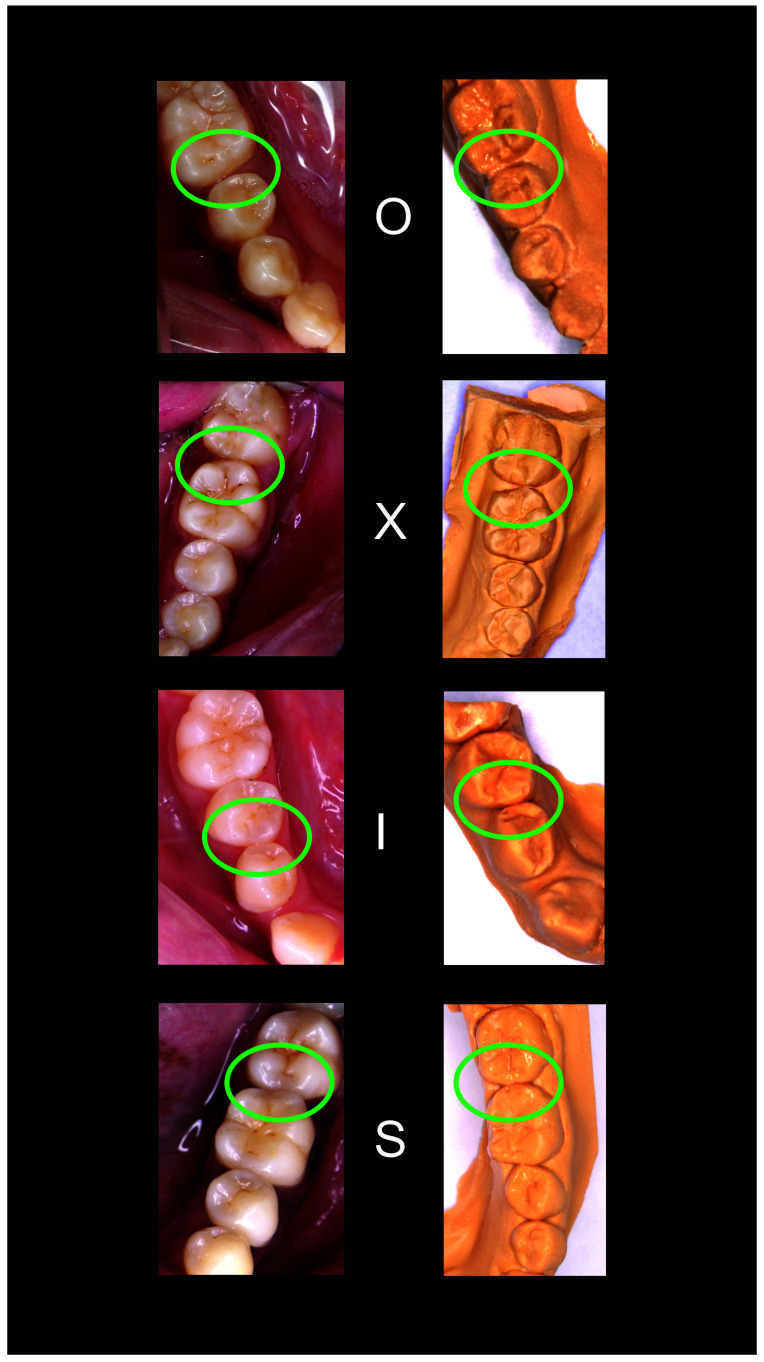
Representative cast images and their equivalent clinical photographs of the interproximal contacts of the mandibular posterior region.

**Figure 5.**
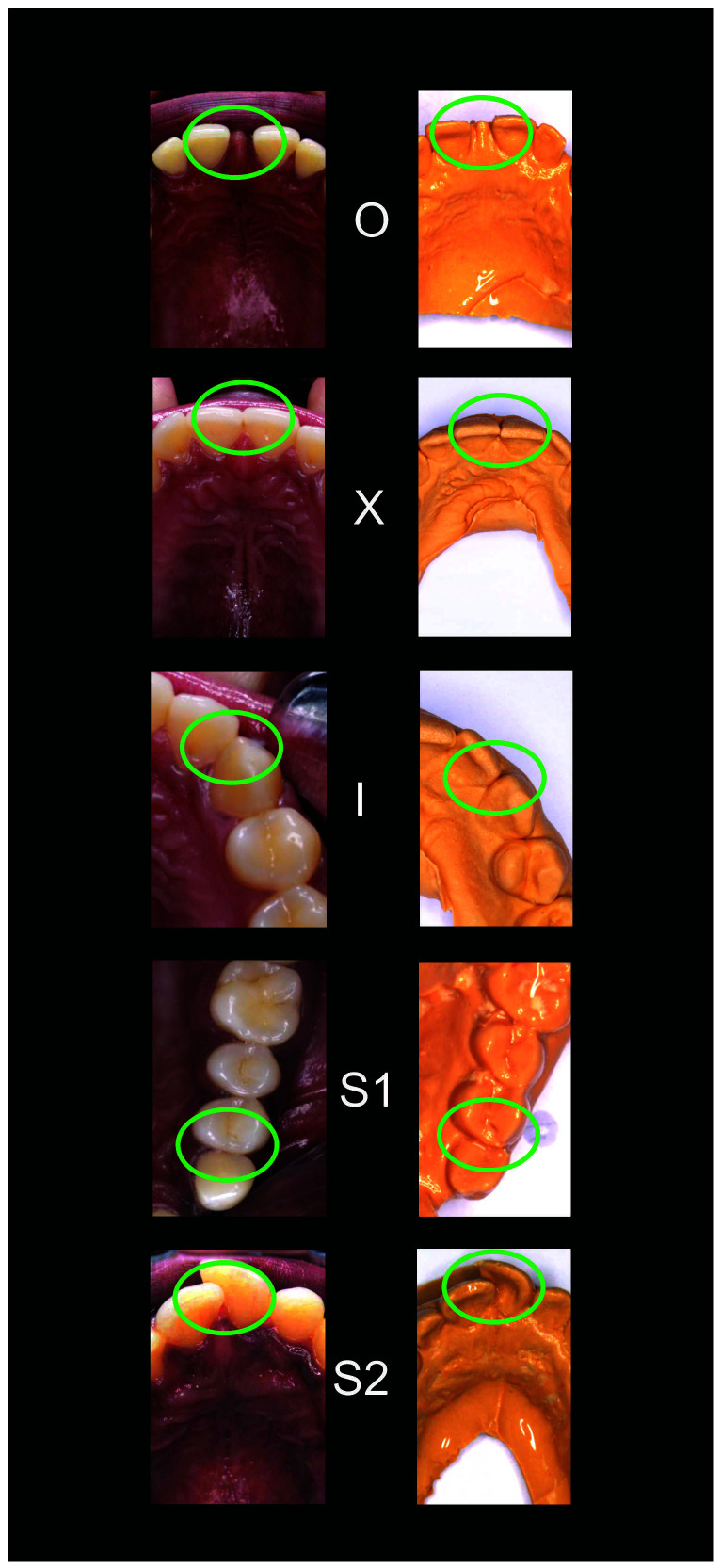
Representative cast images and their equivalent clinical photographs of the interproximal contacts of the maxillary anterior region.

**Figure 6.**
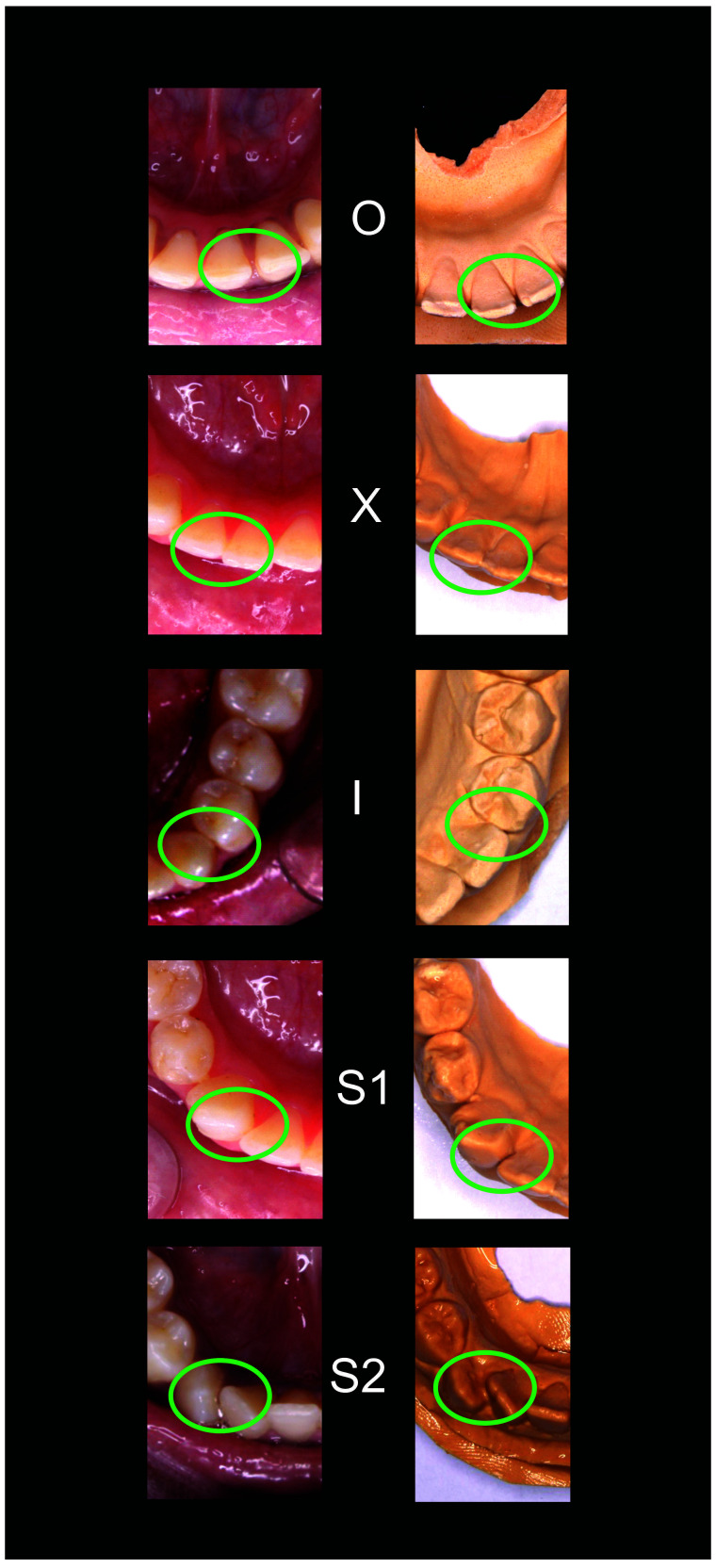
Representative cast images and their equivalent clinical photographs of the interproximal contacts of the mandibular anterior region.

***Statistical analysis*.** Statistical analysis was performed with SPSS 19.0 software (SPSS, Chicago, Ill., USA). Given that SPSS is proprietary, for the purpose of reproducibility, we can recommend PSPP 1.6. 2. GNU PSPP or Microsoft Excel as free alternatives. Numbers and percentages were used to express the prevalence of the types of contact areas. In total, 8918 contacts, that is, 4116 posterior and 4802 anterior contacts were evaluated. To determine the association of contact areas in each quadrant across gender chi square test was applied. McNemar’s test was used to assess intra- and interarch variability. A *P* value of less than 0.05 was deemed to indicate statistical significance. 

## Results

### Prevalence and percentages

Of the 343 patients, 190 were female and 153 were male, with ages ranging from 14 years and 3 months to 23 years and 9 months (mean age, 20 years and 3 months). Table 1– Table 3 summarize the prevalence and percentages of contacts in the permanent dentition according to arch, side, and gender. Among the posterior contacts, the ‘O’ type was the least prevalent, while the ‘S’ type was the most prevalent among the second molar-first molar contacts, the ‘I’ type among the first molar-second premolar contacts, and the ‘X’ type among the second premolar-first premolar contacts. Among the anterior contacts, the least prevalent was the ‘S1’ type for the first premolar-canine contact, and the ‘I’ type for the canine-lateral incisor contact and the lateral incisor-central incisor contact. The most prevalent was the ‘X’ type among all anterior contacts except the canine-lateral incisor contact, where the ‘S2’ type was most prevalent. There was no statistical significance between the right- and left-side contacts ( *P* > 0.05). However, statistical significance was seen between the maxillary and mandibular contacts ( *P* < 0.05).

**Table 1.** Prevalence and percentages of posterior teeth contacts by gender, arch and side.

	*Type of* *Contact*	*Maxilla (N = 686)*						*Mandible (N = 686)*						*Overall* *(N = 1372)*		P *Value,* *Maxilla vs* *Mandible*
		*Right* *(N = 343)*		*Left* *(N = 343)*		*Total*	*%*	*Right* *(N = 343)*		*Left* *(N = 343)*		*Total*	*%*	*Total*	*%*	
		*M*	*F*	*M*	*F*			*M*	*F*	*M*	*F*					
Second molar- first molar contact	O	2	1	1	0	4	0.58	0	0	0	0	0	0	4	0.29	0.04
	X	17	24	11	18	70	10.20	42	67	66	66	241	35.13	311	22.67	0.00
	I	37	47	29	47	160	23.32	68	66	53	72	259	37.78	419	30.54	0.00
	S	97	118	112	125	452	65.89	43	57	34	52	186	27.11	638	46.50	0.00
	Total	153	190	153	190	686	100	153	190	153	190	686	100	1372	100	
	Gender vs right maxilla: χ2 = 0.79, *P* = 0.85. Gender vs left maxilla: χ2 = 3.72, *P* = 0.29. Gender vs right mandible: χ2 = 3.78, *P* = 0.29. Gender vs left mandible: χ2 = 2.70, *P* = 0.44.															
First molar- second premolar contact	O	2	2	0	2	6	0.87	2	2	0	2	6	0.87	12	0.87	1
	X	61	75	69	85	290	42.27	35	55	49	67	206	30.03	496	36.15	0.00
	I	69	88	70	83	310	45.19	76	81	77	86	320	46.65	630	45.92	0.69
	S	21	25	14	20	80	11.66	40	52	27	35	154	22.45	234	17.05	0.00
	Total	153	190	153	190	686	100	153	190	153	190	686	100	1372	100
	Gender vs right maxilla: χ2 = 0.10, *P* = 0.99. Gender vs left maxilla: χ2 = 1.86, *P* = 0.60. Gender vs right mandible: χ2 = 2.20, *P* = 0.53. Gender vs left mandible: χ2 = 2.36, *P* = 0.50.															
Second premolar- first premolar contact	O	6	5	4	6	21	3.06	5	2	2	2	11	1.60	32	2.33	0.08
	X	69	97	87	107	360	52.48	89	120	89	111	409	59.62	769	56.04	0.07
	I	71	84	60	75	290	42.27	53	62	59	67	241	35.13	531	38.70	0.03
	S	7	4	2	2	15	2.19	6	6	3	10	25	3.64	40	2.92	0.11
	Total	153	190	153	190	686	100	153	190	153	190	686	100	1372	100	
	Gender vs right maxilla: χ2 = 2.76, *P* = 0.43. Gender vs left maxilla: χ2 = 0.14, *P* = 0.98. Gender vs right mandible: χ2 = 2.63, *P* = 0.45. Gender vs left mandible: χ2 = 2.74, *P* = 0.43.															

**Table 2.** Prevalence and percentages of anterior teeth contacts by gender, arch and side.

	*Type* *of Contact*	*Maxilla (N = 686)*						*Mandible (N = 686)*						*Overall* *(N = 1372)*		P *Value,* *Maxilla vs* *Mandible*
		*Right* *(N = 343)*		*Left* *(N = 343)*		*Total*	*%*	*Right* *(N = 343)*		*Left* *(N = 343)*		*Total*	*%*	*Total*	*%*	
		*M*	*F*	*M*	*F*			*M*	*F*	*M*	*F*					
First premolar-canine contact	O	47	39	44	37	167	24.34	30	15	27	19	91	13.26	258	18.80	0.00
	X	59	46	54	53	212	30.90	65	76	61	72	274	39.94	486	35.42	0.00
	I	26	59	30	56	171	24.92	16	31	19	29	95	13.85	266	19.39	0.00
	S1	12	35	13	34	94	13.70	10	24	9	22	65	9.48	159	11.59	0.00
	S2	9	11	12	10	42	6.12	32	44	37	48	161	23.47	203	14.80	0.02
	Total	153	190	153	190	686	100	153	190	153	190	686	100	1372	100	
	Gender vs right maxilla: χ2 = 22.89, *P* = 0.00. Gender vs left maxilla: χ2 = 13.33, *P* = 0.00. Gender vs right mandible: χ2 = 14.29, *P* = 0.00. Gender vs left mandible: χ2 = 7.31, *P* = 0.06.															
Canine-lateral incisor contact	O	63	66	66	63	258	37.60	20	12	19	16	67	9.77	325	19.31	0.00
	X	13	22	3	23	61	8.89	6	10	7	9	32	4.66	93	6.78	0.00
	I	4	4	16	7	31	4.52	7	4	4	10	25	3.64	56	4.08	0.42
	S1	46	66	26	62	200	29.15	49	78	57	67	251	36.59	451	32.87	0.01
	S2	27	32	42	35	136	19.83	71	86	66	88	311	45.33	447	32.58	0.00
	Total	153	190	153	190	686	100	153	190	153	190	686	100	1372	100	
	Gender vs right maxilla: χ2 = 2.38, *P* = 0.50. Gender vs left maxilla: χ2 = 27.62, *P* = 0.00. Gender vs right mandible: χ2 = 7.95, *P* = 0.04. Gender vs left mandible: χ2 = 2.88, *P* = 0.41															
Lateral incisor-central incisor contact	O	50	56	49	58	213	31.05	12	22	17	19	70	10.20	283	20.63	0.00
	X	53	67	39	59	218	31.78	69	96	63	72	300	43.73	518	37.76	0.08
	I	13	11	2	16	42	6.12	11	5	12	11	39	5.69	81	5.90	0.74
	S1	4	6	10	6	26	3.79	12	6	10	18	46	11.91	72	5.25	0.01
	S2	33	50	53	51	187	27.26	49	61	51	70	231	33.67	418	30.47	0.03
	Total	153	190	153	190	686	100	153	190	153	190	686	100	1372	100	
	Gender vs right maxilla: χ2 = 1.26, *P* = 0.74. Gender vs left maxilla: χ2 = 11.22, *P* = 0.01. Gender vs right mandible: χ2 = 9.03, *P* = 0.03. Gender vs. left mandible: χ2 = 1.76, *P* = 0.62.															

**Table 3.** Prevalence and percentages of midline teeth contacts by gender, arch and side.

*Type of* *contact*	*Maxilla (N = 343)*				*Mandible (N = 343)*				*Overall* *(N = 686)*		P *Value,* *Maxilla vs* *Mandible*	*Chi and* P *Values,* *Gender vs* *Maxilla*	*Chi and* P *Values,* *Gender vs* *Mandible*
	*Midline*		*Total*	*%*	*Midline*		*Total*	*%*	*Total*	*%*			
	*M*	*F*			*M*	*F*							
O	51	63	114	33.23	22	19	41	11.95	155	22.60	0.00	χ ^2^ = 0.62 0.89	χ ^2^ = 2.75 0.43
X	62	79	141	41.11	82	101	183	53.35	324	47.23	0.00		
I	12	11	23	6.71	9	18	27	7.87	50	7.29	0.57		
S1	9	10	19	5.54	12	15	27	7.87	46	6.70	0.24		
S2	19	27	46	13.41	28	37	65	18.95	111	16.18	0.71		

### Similarities of contacts

The similarities of contacts in all four quadrants in the posterior segment were 5.17% for the second molar-first molar contacts, 5.47% for the first molar-second premolar contacts, and 8.02% for the second premolar-first premolar contacts. The similarities of contacts in all four quadrants in the anterior segment were 5.32% for the premolar-canine contact, 4.08% for the canine-lateral incisor contact, and 4.88% for the lateral incisor-central incisor contact. When the midline contacts were assessed, 10.05% of the samples had similar contacts in the maxilla and the mandible. 

### Intra-arch, Interarch, and Gender Comparisons

When the intra-arch comparison (right vs left) was performed for the same individual, there was a statistically significant difference ( *P* < 0.05) for the ‘X’, ‘I’, and ‘S’ types of contacts on the right and left sides for second molar-first molar contacts, while there was a statistically significant difference for all four contact types for first molar-second premolar contacts ( *P* < 0.05). No statistically significant associations were found for the posterior and anterior contacts when gender was compared in individual quadrants ( *P* >0.05), except for the first premolar-canine contacts (gender vs right and left maxilla and right mandible) and for the canine-lateral incisor contacts (gender vs left maxilla) ( *P* < 0.05).

## Discussion

While Andrews’ six keys to occlusion are the objectives of successful orthodontic treatment ^1^, there is only minimal literature on the fifth key, that is, interproximal contacts. Previous literature on the primary dentition has described the contacts as open/closed ^9– 14^ or as convex and concave ^7^. To the best of the authors’ knowledge, there is no existing classification for inter proximal contacts of the permanent teeth. The impetus for this proposed classification was based on the sequence of manuscripts on OXIS contacts and their reports on the association/susceptibility of various patterns of contacts to caries ^2, 3, 15– 18^. This present retrospective study of ours assessed the interproximal contact variations of permanent teeth and aimed to propose a classification which would be simple yet comprehensive.

The prevalence of the contacts revealed that the broader contacts, that is, the ‘I’ and ‘S’ types, were predominantly associated with the posterior contacts, while the anterior contacts were more of the S1 and S2 types. The ‘O’ type of contact was more commonly seen in the maxilla and warrants further research. While there was minimal difference between gender in the types of posterior contacts, there was an alteration in the distribution of the types of contacts between the maxilla and the mandible. This could not be compared with other studies due to a lack of previous data available in the literature. Approximately 90% of the patients had more than one type of contact in different quadrants. This would indicate that the contact is established by local factors and is an unexplored aspect which may be critical in the development of single or random caries lesions.

The types of contacts could play a substantial role in plaque buildup and caries development. When compared with the ‘O’ and ‘X’ types the ‘I’ and ‘S’ types of contacts would be inaccessible for mechanical cleansing ^2^. Muthu *et al.*, in their retrospective cohort study evaluated the caries risk of the contacts of primary molars based on the OXIS classification and reported that the S and I types of contacts had higher odds of caries development compared with the X and O types ^18^. Not only would orthodontic treatment alter the contacts, but also procedures like interproximal reduction and extractions would alter the contact and its type. Therefore, future studies should focus on the long-term stability of the types of contacts following orthodontic treatment.

Stappert *et al.* ^19^ reported that the quantum of the contact area decreased progressively from 4mm at the midline contact to 1.5mm at the canine premolar contact. They reported that contact areas, not contact points, were observed between neighbouring maxillary anterior teeth. The findings of our study were not in consonance with Stappert *et al.* ^20^ While our study had a greater prevalence of “X” contacts, and therefore point contacts, the reason for this variation could be because Stappert *et al.* ^19^ assessed the interproximal contacts on patients who had no crowding or spacing in the anterior teeth. Thus, it would not be representative of our sample which had malocclusion and therefore malaligned teeth. Further, our study was a linear measurement based on visual observation of the interproximal contact while Stappert *et al.* ^19^ quantified the area based on mathematical calculations.

By far, the most significant orthodontic clinical implication of the type of contact area, especially with fixed appliance therapy is that it could be a determining factor for the development of proximal caries. This has been further established in controversial literature regarding the relationship between dental caries and orthodontic treatment, in that, while orthodontic treatment has been implicated in alteration of the oral environment by providing retention sites for dental plaque ^20^ and therefore increasing the risk of caries development ^21– 23^, other authors believe that fixed orthodontic appliances may not be the singular factor in the development of caries in the patient undergoing fixed orthodontic treatment ^24, 25^. It could therefore be considered that the type of contact would make a difference in this varying literature. It is plausible to hypothesize that teeth could be at higher risk for caries formation in patients with fixed appliances where broader contacts (‘I’ and ‘S’ types) and rotated teeth (‘S1’ and ‘S2’) are present.

The strengths of our study are that it provides the variations in the Andrews’ fifth key i.e. inter proximal contacts of permanent teeth for the first time in the literature and it has assessed the types of contact areas in permanent teeth with a large sample of 8918 intact interproximal contacts and classified them according to OXIS criteria. A major limitation is that the sample was obtained from patients with malocclusion and hence may not be reflective of the general population.

The directions of future research are the following:

- Longitudinal assessment of the change in interproximal contacts with age and eruption of the third molars need to be studied.
- Stability/Relapse of the treated occlusion with different interproximal contact types.
- Incidence of proximal caries with different interproximal contact types in the permanent dentition and in patients undergoing orthodontic treatment.
- Variation in interproximal contacts in different malocclusions (namely Class I, Class II, Class III etc.) and different ethnic populations.

## Conclusion

The OXIS classification of interproximal contacts is applicable to posterior and anterior permanent teeth. Among the posterior contacts, the ‘O’ type of contact was the least prevalent, while ‘I’ type was the most prevalent. Among the anterior contacts, the ‘I’ type of contact was the least prevalent, while the ‘X’ type was the most prevalent. No significant associations were found for the contacts when gender was compared in individual quadrants ( *P* > 0.05). Similarity of contact in all four quadrants ranged from 5.17% to 10.05%.

## Data Availability

Open Science Framework: An evaluation of the interproximal contacts of the permanent dentition-A study cast based classification. [DOI https://doi.org/10.17605/OSF.IO/3CDGN] ^26^ This project contains the following underlying data: Wellcome trust final 05022023 (A study cast classification). Data is available under the terms of the Data are available under the terms of the Creative Commons Zero “No rights reserved” data waiver (CC0 1.0 Public domain dedication).
