# Supplementary material for: Prevalence of different types of interproximal contacts in the permanent dentition – a study cast evaluation
Source: Wellcome Open Res. 2024 Aug 19;8:176. Originally published 2023 Apr 18. [Version 3] doi: 10.12688/wellcomeopenres.18973.3 (PMC11234082; doi:10.12688/wellcomeopenres.18973.3)
Supplement: Peer review history

# Reviewer report: Reviewer response for version 3

**DOI:** 10.21956/wellcomeopenres.25170.r115977

**Published:** 2025-01-07

**Authors:** Maria-Angelica Bencze

**Affiliations:** 1 “Carol Davila” University of Medicine and Pharmacy, Bucharest, Romania

Dear Authors,

 Congratulations on your work! I think your study is valuable research, well written and with clinical significance.

 In the Discussion section, I would comment the type of anterior teeth contact points related to incisors morphology. The casts with malocclusions (included in your study group) present often different degrees of crowding. As previously noted, the crowding cases frequently present upper incisors with decreased buco-lingual diameters and larger crown proportions, compared with well aligned arches. These morphological particularities may influence the type of interproximal contacts.

 Except this comment, I appreciate that the document is suitable for Indexing.

Is the work clearly and accurately presented and does it cite the current literature?

Yes

If applicable, is the statistical analysis and its interpretation appropriate?

Yes

Are all the source data underlying the results available to ensure full reproducibility?

Yes

Is the study design appropriate and is the work technically sound?

Yes

Are the conclusions drawn adequately supported by the results?

Yes

Are sufficient details of methods and analysis provided to allow replication by others?

Yes

Reviewer Expertise:

orthodontics, temporomandibular disorders, craniofacial development

I confirm that I have read this submission and believe that I have an appropriate level of expertise to confirm that it is of an acceptable scientific standard.

## References

[1] : Implications of Permanent Teeth Dimensions and Arch Lengths on Dental Crowding during the Mixed Dentition Period. *Applied Sciences* .2021;11(17) : 10.3390/app11178004 10.3390/app11178004

# Reviewer report: Reviewer response for version 3

**DOI:** 10.21956/wellcomeopenres.25170.r113263

**Published:** 2025-01-03

**Authors:** Manoj Jaiswal

**Affiliations:** 1 Postgraduate Institute of Medical Education and Research, Chandigarh, India

- A well-written article with appropriate methodology and statical analysis, reference the research paper.

- Focuses on the type of contact and their applicability in future caries prediction.
- A modification can be made in accessing the proximal contact, which needs to be made to use digital tools to access them so that subjective error can be avoided.
- The clinical applicability can be explored more.

Is the work clearly and accurately presented and does it cite the current literature?

Yes

If applicable, is the statistical analysis and its interpretation appropriate?

Yes

Are all the source data underlying the results available to ensure full reproducibility?

Yes

Is the study design appropriate and is the work technically sound?

Yes

Are the conclusions drawn adequately supported by the results?

Yes

Are sufficient details of methods and analysis provided to allow replication by others?

Yes

Reviewer Expertise:

sleep apnea, dental caries, genral anesthesia, application of AI In dentistry

I confirm that I have read this submission and believe that I have an appropriate level of expertise to confirm that it is of an acceptable scientific standard.

# Reviewer report: Reviewer response for version 3

**DOI:** 10.21956/wellcomeopenres.25170.r108366

**Published:** 2024-11-08

**Authors:** Hatem Saifeldin

**Affiliations:** 1 Orthodontics, Ain Shams University, Cairo, Cairo Governorate, Egypt

Dear Authors,

 Thank you for submitting this article. I would like you to,

1. Include a null hypothesis in the introduction section.
2. Please add this to the title: Prevalence of different types of interproximal contacts in the permanent dentition – a retrospective cross-sectional study cast evaluation.
3. More citations can help you.

Is the work clearly and accurately presented and does it cite the current literature?

Yes

If applicable, is the statistical analysis and its interpretation appropriate?

Yes

Are all the source data underlying the results available to ensure full reproducibility?

Yes

Is the study design appropriate and is the work technically sound?

Yes

Are the conclusions drawn adequately supported by the results?

Yes

Are sufficient details of methods and analysis provided to allow replication by others?

Yes

Reviewer Expertise:

Orthodontics

I confirm that I have read this submission and believe that I have an appropriate level of expertise to confirm that it is of an acceptable scientific standard, however I have significant reservations, as outlined above.

## References

[1] : Evaluation of digital and manual orthodontic diagnostic setups in non-extraction cases using ABO model grading system: an in-vitro study. *BMC Oral Health* .2024;24(1) : 10.1186/s12903-024-03961-z 207 10.1186/s12903-024-03961-z 38336704 PMC10858554

# Reviewer report: Reviewer response for version 3

**DOI:** 10.21956/wellcomeopenres.25170.r93997

**Published:** 2024-09-09

**Authors:** Baranya Shrikrishna Suprabha

**Affiliations:** 1 Manipal Academy of Higher Education, Manipal, Karnataka, India

The authors have responded to the  reviewer  comments convincingly and comprehensively. I do not have any further suggestions for the manuscript.

Is the work clearly and accurately presented and does it cite the current literature?

Partly

If applicable, is the statistical analysis and its interpretation appropriate?

Yes

Are all the source data underlying the results available to ensure full reproducibility?

Yes

Is the study design appropriate and is the work technically sound?

Partly

Are the conclusions drawn adequately supported by the results?

Yes

Are sufficient details of methods and analysis provided to allow replication by others?

Partly

Reviewer Expertise:

early childhood caries

I confirm that I have read this submission and believe that I have an appropriate level of expertise to confirm that it is of an acceptable scientific standard.

# Reviewer report: Reviewer response for version 2

**DOI:** 10.21956/wellcomeopenres.22451.r86450

**Published:** 2024-07-10

**Authors:** Baranya Shrikrishna Suprabha

**Affiliations:** 1 Manipal Academy of Higher Education, Manipal, Karnataka, India

While I appreciate the novelty of the study objective, application of OXIS classification in permanent dentition, the vivid presentation in the introduction, results and discussion section, some queries remain regarding the methods section:

1. In the inclusion criteria, add that the casts were of patients seeking treatment for malocclusion, if pre-treatment casts were used. Please specify whether pre-treatment/post-treatment casts were used.
2. While the code S2 is designated for anterior teeth contact with minor rotation, how was the coding considered in case of mild rotation of posterior teeth?
3. “Whenever there was any doubt regarding the quantum of measurement with visual inspection, a Williams probe was used to measure the distance and the contact was graded accordingly.” The description of measurement needs to be more specific. How was 1.5mm contact (As per OXIS) measurement done just by visual examination, without measuring each cast?
4. How was the accuracy of the examiner performing visual examination on orthodontic casts and classifying the contacts ensured? How was the reliability ensured? Was any calibration done with an expert examiner? Please provide inter and intra-rater reliability values.
5. Were the dental casts anonymized during measurement? How was the examiner bias minimized regarding gender and age variation of the patients whose dental casts were examined?
6. I understand that the use of dental casts of patients with malocclusion is a major study limitation. Please add in discussion, the applicability of the study findings (in terms of prevalence of various contacts obtained in the study)
7. In the introduction, the sentence “The variations in the contact areas of the permanent dentition might alter caries susceptibility and have orthodontic implications affecting the magnitude of force application, force distribution from one tooth to another and stability of orthodontic treatment” requires a reference.

Is the work clearly and accurately presented and does it cite the current literature?

Partly

If applicable, is the statistical analysis and its interpretation appropriate?

Yes

Are all the source data underlying the results available to ensure full reproducibility?

Yes

Is the study design appropriate and is the work technically sound?

Partly

Are the conclusions drawn adequately supported by the results?

Yes

Are sufficient details of methods and analysis provided to allow replication by others?

Partly

Reviewer Expertise:

early childhood caries

I confirm that I have read this submission and believe that I have an appropriate level of expertise to confirm that it is of an acceptable scientific standard, however I have significant reservations, as outlined above.

# Response

**Published:** 2024-07-20

**Authors:** M KIRTHIGA

**Affiliations:** Dr., India

1. In the inclusion criteria, add that the casts were of patients seeking treatment for malocclusion, if pre-treatment casts were used. Please specify whether pre-treatment/post-treatment casts were used.

**Response:** We thank the Reviewer for this suggestion. We have modified the sentence by specifying that pre treatment casts were used. From Inclusion and exclusion criteria. The inclusion criteria were: casts of patients with intact contacts, with a full complement of permanent teeth other than third molars, and between the ages of 14 years and 25 years. To Inclusion and exclusion criteria. The inclusion criteria were: pretreatment casts of patients with intact contacts, with a full complement of permanent teeth other than third molars, and between the ages of 14 years and 25 years.

1. While the code S2 is designated for anterior teeth contact with minor rotation, how was the coding considered in case of mild rotation of posterior teeth?

**Response:** While we are grateful to the Reviewer for this query, the rotations of posterior teeth were covered within the OXIS classification. Posterior teeth with broader contact were observed to have S and I type contacts while those with rotations had X type contacts. No changes made.

1. “Whenever there was any doubt regarding the quantum of measurement with visual inspection, a Williams probe was used to measure the distance and the contact was graded accordingly.” The description of measurement needs to be more specific. How was 1.5mm contact (As per OXIS) measurement done just by visual examination, without measuring each cast?

**Response:** While we appreciate the intent of the Reviewer to strengthen the assessment methodology, we would like to reinforce that we used Williams probe only for the borderline cases which could not be categorized with certainty on visual inspection. No changes made.

1. How was the accuracy of the examiner performing visual examination on orthodontic casts and classifying the contacts ensured? How was the reliability ensured? Was any calibration done with an expert examiner? Please provide inter and intra-rater reliability values.

**Response:** We acknowledge the query of the Reviewer. The inter examiner variability a part of the training and calibration process and was 0.92. The reliability was ensured by training and  calibration with an expert examiner. The intra examiner reliability data has been provided in the manuscript. This information is available in the paragraph) titled “Training and calibration of the examiner.” (Page 4 -appended  and highlighted below for the Reviewer’s convenience). Before the study began in the university orthodontic department, a single dentist VK was extensively trained and calibrated under the supervision of one of the authors of the OXIS classification, to evaluate the contact areas. The calibration process involved a presentation of the classification and theoretical discussions on its use, followed by practical sessions thereon. Finally, a clinical exercise involving examination and re-examination of the same 20 patient study casts (26 intact contacts per patient), comprising 520 intact contacts, was screened by the same primary investigator on two separate occasions after a three-week interlude. The kappa value to test the intra-examiner variability was obtained as 0.82, which reflected a high degree of agreement. No changes made. 

1. Were the dental casts anonymized during measurement? How was the examiner bias minimized regarding gender and age variation of the patients whose dental casts were examined?

**Response:** We are obliged to the Reviewer for the query. The Primary Investigator assessed the casts based on the Out Patient Registration number. This enabled anonymization to the patient details like gender and age, thus ensuring minimization of  examiner and observer bias. No changes made.

1. I understand that the use of dental casts of patients with malocclusion is a major study limitation. Please add in discussion, the applicability of the study findings (in terms of prevalence of various contacts obtained in the study)

**Response:** We are obliged to the reviewer for this suggestion. We have modified the Discussion section accordingly. From 'It is plausible to hypothesize that teeth could be at higher risk for caries formation in patients with fixed appliances where broader contacts (‘I’ and ‘S’ types) and rotated teeth (‘S1’ and ‘S2’) are present'. Moreover, contacts would be corrected with orthodontic treatment and it would be of clinical interest to compare these findings with the treated occlusion.

1. In the introduction, the sentence “The variations in the contact areas of the permanent dentition might alter caries susceptibility and have orthodontic implications affecting the magnitude of force application, force distribution from one tooth to another and stability of orthodontic treatment” requires a reference.

**Response:** We are indebted to the reviewer for this suggestion. We have edited the sentence and have added the following references. From 'The variations in the contact areas of the permanent dentition might alter caries susceptibility and have orthodontic implications affecting the magnitude of force application, force distribution from one tooth to another, and stability of orthodontic treatment' to 'With compelling evidence suggesting that variations in contacts between teeth are important in the development of proximal caries in the primary dentition ^8-10^, it would be of clinical interest to assess it in the permanent dentition. The variations could have orthodontic implications like the stability of orthodontic treatment ^11^, magnitude of force application and force distribution from one tooth to another since the interface between adjacent teeth is due to an equilibrium between the compressive force that each tooth exerts on its counterpart and the resisting force which tries to separate them ^12^.'  References added 8. Muthu MS, Kirthiga M, Lee JC, et al.: OXIS contacts as a risk factor for approximal caries: a retrospective cohort study. *Pediatr Dent.* 2021; 43: 296–300. 344678477612504 9. Kirthiga M, Muthu MS, Kayalvizhi G, et al.: Variations in Primary Molar Contact and Approximal Caries in Children: A Three-Year Prospective Cohort Study. *Pediatr Dent.* 2023;45:434-442. 37904267 10. Kirthiga M, Muthu MS, Kayalvizhi G, et al: OXIS contacts and approximal caries in preschool children- A prospective cohort study. *Caries Res.* 2023;57:133–140. 10.1159/000529160 11. Christine A, Eliades T, Spyridon N. Stability of occlusal outcome during long-term retention: the time-dependent variation of the American Board of Orthodontics index. *Eur J Orthod.* 2021; 43(1):1-7. 10.1093/ejo/cjaa004 12. Vardimon AD, Matsaev E, Lieberman M, Brosh T. Tightness of dental contact points  in spaced and  non-spaced permanent dentitions. *Eur J Orthod.* 2001;23:305-314. 10.1093/ejo/23.3.305

# Reviewer report: Reviewer response for version 2

**DOI:** 10.21956/wellcomeopenres.22451.r69197

**Published:** 2023-11-21

**Authors:** Tarulatha R. Shyagali

**Affiliations:** 1 Orthodontics and Dentofacial Orthopedics, M R Ambedkar Dental College and Hospital, Bengaluru, Karnataka, India

I feel the manuscript requires no further changes.

Is the work clearly and accurately presented and does it cite the current literature?

Yes

If applicable, is the statistical analysis and its interpretation appropriate?

Yes

Are all the source data underlying the results available to ensure full reproducibility?

Partly

Is the study design appropriate and is the work technically sound?

Partly

Are the conclusions drawn adequately supported by the results?

Yes

Are sufficient details of methods and analysis provided to allow replication by others?

Partly

Reviewer Expertise:

Orthodontics and Dentofacial Orthopedics

I confirm that I have read this submission and believe that I have an appropriate level of expertise to confirm that it is of an acceptable scientific standard.

# Reviewer report: Reviewer response for version 1

**DOI:** 10.21956/wellcomeopenres.21035.r65764

**Published:** 2023-09-08

**Authors:** Shalini Garg

**Affiliations:** 1 Pediatric and Preventive Dentistry, Shree Guru Gobind Singh Tricentenary University, Gurugram, Haryana, India

1. Sample was taken from a specific group of subject requiring orthodontic treatment.(BIASED)
2. Type of malocclusion and molar relationship will affect type of inter-proximal contacts. It can not be applied to all population groups.
3. Presence absence and eruption status of permanent third molar will affect type of contacts depending upon age of the patient.
4. Presence of hidden inter-proximal caries can affect the OXIS classification specially with broader contact area.
5. The inclusion and exclusion criteria need to be thoroughly revised .
6. The exact measurement of less or more than 1.5 mm doesn't  seem possible with naked eyes.

Is the work clearly and accurately presented and does it cite the current literature?

Partly

If applicable, is the statistical analysis and its interpretation appropriate?

I cannot comment. A qualified statistician is required.

Are all the source data underlying the results available to ensure full reproducibility?

Partly

Is the study design appropriate and is the work technically sound?

Partly

Are the conclusions drawn adequately supported by the results?

Partly

Are sufficient details of methods and analysis provided to allow replication by others?

Partly

Reviewer Expertise:

Pediatric dentistry , Caries research, Dental trauma

I confirm that I have read this submission and believe that I have an appropriate level of expertise to confirm that it is of an acceptable scientific standard, however I have significant reservations, as outlined above.

# Response

**Published:** 2023-10-14

**Authors:** M KIRTHIGA

**Affiliations:** Dr., India

**Reviewer 2 Queries**

1. Sample was taken from a specific group of subject requiring orthodontic treatment.(BIASED)

We thank the reviewer for this important observation. We have modified the last paragraph of Discussion, From The possible limitations of this study are that the included samples (study casts) are from the patients who reported for orthodontic treatment and hence would have had increased incidence of malpositions like crowding, spacing, rotations etc. To A major limitation is that the sample was obtained from patients with malocclusion and hence may not be reflective of the general population.

1. a. Type of malocclusion and molar relationship will affect type of inter-proximal contacts.

Kindly refer to the answer given to Question no. 3a of Reviewer 1. b. It can not be applied to all population groups. We are in consonance with the comment of the Reviewer. Therefore, we have added it to the directions of future research. Kindly refer to the answer given to Question no. 3a of Reviewer 1.

1. Presence absence and eruption status of permanent third molar will affect type of contacts depending upon age of the patient.

We admit that it would be scientifically relevant to assess the role of third molar in the change in interproximal contacts. Therefore we have added it to the directions of future research. Kindly refer to the answer given to Question no. 3a of Reviewer 1.  

1. Presence of hidden inter-proximal caries can affect the OXIS classification specially with broader contact area.

While we thank the reviewer for this observation, based on the research teams prior experience on OXIS studies on primary teeth ^1,2^ where bite wing radiographs were also used to assess the presence of caries, hidden caries did not contribute to change in the type of interproximal contact. However, if the hidden caries progresses to cavitation (Class II), scoring of the contact type becomes challenging. References

1. Variations in primary molar contact and approximal caries in children: A 3-year prospective cohort study. Kirthiga M, Muthu MS, Kayalvizhi G, Mathur VP, Jayakumar N. Pediatr Dent 2023;45(5):434-9.E48-E50.
2. OXIS contacts and approximal caries in preschool children- A prospective cohort study. Kirthiga M, Muthu MS, Kayalvizhi G, Mathur VP Naveenkumar J, Praveen R. OXIS contacts and approximal caries in preschool children- A prospective cohort study. Caries Res. 2023 January 23; 57(2): 133–140.

 

1. The inclusion and exclusion criteria need to be thoroughly revised .

The inclusion and exclusion criteria have been revised. Kindly refer to the answer given to Question no. 6b of Reviewer 1.  

1. The exact measurement of less or more than 1.5 mm doesn't  seem possible with naked eyes.

We are in concurrence with the observation of the Reviewer. Kindly refer to the answer given to Question no. 2 of Reviewer 1.

# Reviewer report: Reviewer response for version 1

**DOI:** 10.21956/wellcomeopenres.21035.r58270

**Published:** 2023-06-13

**Authors:** Tarulatha R. Shyagali

**Affiliations:** 1 Orthodontics and Dentofacial Orthopedics, M R Ambedkar Dental College and Hospital, Bengaluru, Karnataka, India

Thank you very much for asking me to review this paper, I congratulate the authors for their efforts in pursuing this research, although paper is well written, I have a few queries to make related to this paper; 

1. The authors mention that they used the standardized distance for measurement, could they specify how much is the standardized distance and how did they manage to achieve the same since they were viewing the casts from top/occlusal view? Kindly provide the photo of the assessment if possible. 
2. Since quantifying less than 1.5 and more than 1.5 is so difficult, how did authors manage to achieve it by visual inspection, was there a possibility of using any tool to overcome this drawback? 
3. Although the authors mention that the study was flawed in taking up the cast from department of orthodontics, But I still would be commenting on the fact that, authors could have set the stringent inclusion criteria, so that they could have selected near to ideal or normal occlusion casts. Because slippage of the contact is so common in malocclusion casts. Further, authors give S1 and S2 type of contacts in anterior teeth, which is commendable, however again quantifying the same will be difficult. 
4. Apart from this, if we know what is normally seen in ideal or normal occlusion we could be better prepared to keep the normal contacts intact and treat the abnormal contacts or take precaution to prevent the unwanted side effects from them. 
5. Rather the classification, study was more of the assessment of the prevalence of different types of interproximal contacts. So, I feel authors should take a call on changing the title of the article to suit their study results 
6. Inclusion and exclusion criteria were not set properly, as following major criteria were not taken into consideration; 
  a. Severity of the malocclusion determines the interproximal contact area type, authors failed to set the selection criteria for this. 
  b. A filled or carious or traumatic tooth would obliviously affect the interproximal contact point, authors fail to consider this as well. 
  c. Was there in criteria set to exclude periodontally compromised patients? would this have any affect on your findings. 
7. Will the shape of tooth determine the types of interproximal contact area, was there any consideration given to this factor?
8. Authors finding suggests that, the prevalence for 'x' type contact was more in midline teeth, lateral incisors and central incisors, but article by Stappert et al., suggests that "Contact areas, not contact points, were observed between neighboring maxillary anterior" teeth. ( *Stappert, Christian & Tarnow, Dennis & Tan-Chu, Jocelyn & Chu, Stephen. (2010). Proximal Contact Areas of the Maxillary Anterior Dentition. The International journal of periodontics & restorative dentistry. 30. 471-7*.). Authors can give their feedback on the same.

Is the work clearly and accurately presented and does it cite the current literature?

Yes

If applicable, is the statistical analysis and its interpretation appropriate?

Yes

Are all the source data underlying the results available to ensure full reproducibility?

Partly

Is the study design appropriate and is the work technically sound?

Partly

Are the conclusions drawn adequately supported by the results?

Yes

Are sufficient details of methods and analysis provided to allow replication by others?

Partly

Reviewer Expertise:

Orthodontics and Dentofacial Orthopedics

I confirm that I have read this submission and believe that I have an appropriate level of expertise to confirm that it is of an acceptable scientific standard, however I have significant reservations, as outlined above.

## References

[1] : Proximal contact areas of the maxillary anterior dentition. *Int J Periodontics Restorative Dent* .2010;30(5) :471-7 20814600

# Response

**Published:** 2023-10-14

**Authors:** M KIRTHIGA

**Affiliations:** Dr., India

1. The authors mention that they used the standardized distance for measurement, could they specify how much is the standardized distance and how did they manage to achieve the same since they were viewing the casts from top/occlusal view? Kindly provide the photo of the assessment if possible. We thank the Reviewer for the valuable suggestion and a photo of the assessment has been provided. (Figure 1). The distance was standardized by placing the cast on a specified table. The distance between the eye line of the primary investigator and the cast was measured to be one foot. These standardizations were applied for all measurements.

We have added a new figure, (Figure 1) with appropriate legends. Figure 1: Image depicting standardization of distance during interproximal contact  assessment. Changes made in Materials and Methods, From They were based and placed on a flat table for assessment, and the evaluation was done   from a standardized distance. To They were based and placed on a flat table for assessment, and the evaluation was done from a standardized distance. The distance was standardized by placing the casts on a specified table. The distance between the eye line of the primary investigator and the cast was measured to be one foot. These standardizations were applied for all measurements.  

1. Since quantifying less than 1.5 and more than 1.5 is so difficult, how did authors manage to achieve it by visual inspection, was there a possibility of using any tool to overcome this drawback?  

We are in concurrence with the observation of the Reviewer. Whenever there was any doubt regarding the quantum of measurement with visual inspection, a Williams probe was used to measure the distance and the contact was graded accordingly. Changes  made in Materials and Methods, From The assessment of the maxillary and mandibular casts always began with the second molar-first molar interproximal contact on the right quadrant and proceeded across the midline to the left quadrant second molar-first molar interproximal contact. To The assessment of the maxillary and mandibular casts always began with the second molar-first molar interproximal contact on the right quadrant and proceeded across the midline to the left quadrant second molar-first molar interproximal contact. Whenever there was any doubt regarding the quantum of measurement with visual inspection, a Williams probe was used to measure the distance and the contact was graded accordingly.  

1. a. Although the authors mention that the study was flawed in taking up the cast from department of orthodontics, But I still would be commenting on the fact that, authors could have set the stringent inclusion criteria, so that they could have selected near to ideal or normal occlusion casts. 

This suggestion by the reviewer would have definitely strengthened the inclusion criteria. However, since we planned a separate study for assessing the interproximal contacts in normal occlusion casts, it was not part of the eligibility criteria.  It has been incorporated into the directions of future research. Changes made in Discussion, From Future research should involve longitudinal studies to evaluate the stability/relapse as well as the incidence of proximal caries in patients undergoing orthodontic treatment based on the OXIS classification for permanent dentition. To The directions of future research are the following:

- Longitudinal assessment of the change in interproximal contacts with age and eruption of the third molars need to be studied.
- Stability/Relapse of the treated occlusion with different interproximal contact types.
- Incidence of proximal caries with different interproximal contact types in the permanent dentition and in patients undergoing orthodontic treatment.
- Variation in interproximal contacts in different malocclusions (namely Class I, Class II, Class III etc.) and different ethnic populations..

  b. Because slippage of the contact is so common in malocclusion casts. Further, authors give S1 and S2 type of contacts in anterior teeth, which is commendable, however again quantifying the same will be difficult.   Although categorising S1 and S2 type of contacts is little more complex than O, X and I, it is doable because S1 contact involves one surface and S2 involves two surfaces. By taking a close look at the interproximal contact, we could identify and define S1 and S2. For the reviewer’s ease, the definition is attached below. ^1^ S1: When anterior tooth is rotated and only one of its surfaces (either proximal surface or labial/lingual surface) is contacting the adjacent tooth S2: When anterior is rotated and has two of its surfaces - proximal surface (mesial/distal) and labial or lingual surface contacting the adjacent tooth Reference 1.Aarthi J, Muthu M, Kirthiga M and Kailasam V. Modified OXIS classification for primary canines. *Wellcome Open Res* 2022, 7:130  

1. Apart from this, if we know what is normally seen in ideal or normal occlusion we could be better prepared to keep the normal contacts intact and treat the abnormal contacts or take precaution to prevent the unwanted side effects from them

  We accept and concur with the Reviewer’s suggestion. We will able to provide an answer to this question with a separate study on ideal or normal occlusion which has been planned. It has been added to the directions of future research. Kindly refer to the answer for Question No. 3a  

1. Rather the classification, study was more of the assessment of the prevalence of different types of interproximal contacts. So, I feel authors should take a call on changing the title of the article to suit their study results.

  We are in concurrence with the Reviewer’s reasoning on the need for Title change. We propose to change the Title,  From “An evaluation of the interproximal contacts of the permanent dentition-A study cast based classification” To “Prevalence of different types of interproximal contacts in the permanent dentition – a study cast evaluation.”  

1. Inclusion and exclusion criteria were not set properly, as following major criteria were not taken into consideration;

1. Severity of the malocclusion determines the interproximal contact area type, authors failed to set the selection criteria for this.

We are grateful to the reviewer for pointing out this important parameter. While we do agree that malocclusion type would have an impact on the type of contacts, we did not consider the malocclusion severity/classification in the eligibility criteria as we had planned a separate study on the prevalence of the various types of interproximal contacts and different malocclusions. Kindly refer to the answer for Question No. 3a

1. A filled or carious or traumatic tooth would obliviously affect the interproximal contact point, authors fail to consider this as well.

We acknowledge and are grateful to the Reviewer for the observation. Though filled, carious and traumatic teeth were not mentioned as part of the exclusion criteria, they were excluded from the study. We have now added them in the exclusion criteria. Changes made in Materials and Methods From The exclusion criteria were any occlusal wear and any developmental anomalies affecting the morphology of the tooth (peg laterals, etc.) To The exclusion criteria were casts of patients with carious, filled or fractured teeth, occlusal wear and  any developmental anomalies affecting the morphology of the tooth (peg laterals, etc.)

1. Was there in criteria set to exclude periodontally compromised patients? would this have any affect on your findings.         

We concede that a compromised periodontium might affect the interproximal contatcts. With the study casts, a periodontal evaluation was difficult. Hence the periodontal status was not considered.   

1. Will the shape of tooth determine the types of interproximal contact area, was there any consideration given to this factor?

We endorse the Reviewer’s question. Yes, the shape of the tooth would affect the contact. However, teeth with anomalies affecting their morphology were part of the exclusion criteria. The exclusion criteria were the presence of caries, any occlusal wear and any developmental anomalies affecting the morphology of the tooth (peg laterals, etc.).  

1. Authors finding suggests that, the prevalence for 'x' type contact was more in midline teeth, lateral incisors and central incisors, but article by Stappert et al., suggests that "Contact areas, not contact points, were observed between neighboring maxillary anterior" teeth. (Stappert, Christian & Tarnow, Dennis & Tan-Chu, Jocelyn & Chu, Stephen. (2010). Proximal Contact Areas of the Maxillary Anterior Dentition. The International journal of periodontics & restorative dentistry. 30. 471-7.). Authors can give their feedback on the same          

We are grateful to the Reviewer for bringing this important study to our attention. The findings of Stappert et al have been added in the “Discussion” section of our manuscript. Changes made to Discussion – (Paragraph added and References changed) Stappert et al ^19^ reported that the quantum of the contact area decreased progressively from 4mm at the midline contact to 1.5mm at the canine premolar contact. They reported that contact areas, not contact points, were observed between neighbouring maxillary anterior teeth. The findings of our study were not in consonance with Stappert et al ^19^. While our study had a greater prevalence of “X” contacts, and therefore point contacts, the reason for this variation could be because Stappert et al ^19^ assessed the interproximal contacts on patients who had no crowding or spacing in the anterior teeth. Thus, it would not be representative of our sample which had malocclusion and therefore malaligned teeth. Further, our study was a linear measurement based on visual observation of the interproximal contact while Stappert et al ^20^ quantified the area based on mathematical calculations.
