# Supplementary material for: Prevalence of different types of interproximal contacts in the permanent dentition – a study cast evaluation
Source: Wellcome Open Res. 2023 Oct 24;8:176. Originally published 2023 Apr 18. [Version 2] doi: 10.12688/wellcomeopenres.18973.2 (PMC11234082; doi:10.12688/wellcomeopenres.18973.2)
Supplement: Peer review history

# Response

**Published:** 2023-10-14

**Authors:** M KIRTHIGA

**Affiliations:** Dr., India

1. The authors mention that they used the standardized distance for measurement, could they specify how much is the standardized distance and how did they manage to achieve the same since they were viewing the casts from top/occlusal view? Kindly provide the photo of the assessment if possible. We thank the Reviewer for the valuable suggestion and a photo of the assessment has been provided. (Figure 1). The distance was standardized by placing the cast on a specified table. The distance between the eye line of the primary investigator and the cast was measured to be one foot. These standardizations were applied for all measurements.

We have added a new figure, (Figure 1) with appropriate legends. Figure 1: Image depicting standardization of distance during interproximal contact  assessment. Changes made in Materials and Methods, From They were based and placed on a flat table for assessment, and the evaluation was done   from a standardized distance. To They were based and placed on a flat table for assessment, and the evaluation was done from a standardized distance. The distance was standardized by placing the casts on a specified table. The distance between the eye line of the primary investigator and the cast was measured to be one foot. These standardizations were applied for all measurements.  

1. Since quantifying less than 1.5 and more than 1.5 is so difficult, how did authors manage to achieve it by visual inspection, was there a possibility of using any tool to overcome this drawback?  

We are in concurrence with the observation of the Reviewer. Whenever there was any doubt regarding the quantum of measurement with visual inspection, a Williams probe was used to measure the distance and the contact was graded accordingly. Changes  made in Materials and Methods, From The assessment of the maxillary and mandibular casts always began with the second molar-first molar interproximal contact on the right quadrant and proceeded across the midline to the left quadrant second molar-first molar interproximal contact. To The assessment of the maxillary and mandibular casts always began with the second molar-first molar interproximal contact on the right quadrant and proceeded across the midline to the left quadrant second molar-first molar interproximal contact. Whenever there was any doubt regarding the quantum of measurement with visual inspection, a Williams probe was used to measure the distance and the contact was graded accordingly.  

1. a. Although the authors mention that the study was flawed in taking up the cast from department of orthodontics, But I still would be commenting on the fact that, authors could have set the stringent inclusion criteria, so that they could have selected near to ideal or normal occlusion casts. 

This suggestion by the reviewer would have definitely strengthened the inclusion criteria. However, since we planned a separate study for assessing the interproximal contacts in normal occlusion casts, it was not part of the eligibility criteria.  It has been incorporated into the directions of future research. Changes made in Discussion, From Future research should involve longitudinal studies to evaluate the stability/relapse as well as the incidence of proximal caries in patients undergoing orthodontic treatment based on the OXIS classification for permanent dentition. To The directions of future research are the following:

- Longitudinal assessment of the change in interproximal contacts with age and eruption of the third molars need to be studied.
- Stability/Relapse of the treated occlusion with different interproximal contact types.
- Incidence of proximal caries with different interproximal contact types in the permanent dentition and in patients undergoing orthodontic treatment.
- Variation in interproximal contacts in different malocclusions (namely Class I, Class II, Class III etc.) and different ethnic populations..

  b. Because slippage of the contact is so common in malocclusion casts. Further, authors give S1 and S2 type of contacts in anterior teeth, which is commendable, however again quantifying the same will be difficult.   Although categorising S1 and S2 type of contacts is little more complex than O, X and I, it is doable because S1 contact involves one surface and S2 involves two surfaces. By taking a close look at the interproximal contact, we could identify and define S1 and S2. For the reviewer’s ease, the definition is attached below. ^1^ S1: When anterior tooth is rotated and only one of its surfaces (either proximal surface or labial/lingual surface) is contacting the adjacent tooth S2: When anterior is rotated and has two of its surfaces - proximal surface (mesial/distal) and labial or lingual surface contacting the adjacent tooth Reference 1.Aarthi J, Muthu M, Kirthiga M and Kailasam V. Modified OXIS classification for primary canines. *Wellcome Open Res* 2022, 7:130  

1. Apart from this, if we know what is normally seen in ideal or normal occlusion we could be better prepared to keep the normal contacts intact and treat the abnormal contacts or take precaution to prevent the unwanted side effects from them

  We accept and concur with the Reviewer’s suggestion. We will able to provide an answer to this question with a separate study on ideal or normal occlusion which has been planned. It has been added to the directions of future research. Kindly refer to the answer for Question No. 3a  

1. Rather the classification, study was more of the assessment of the prevalence of different types of interproximal contacts. So, I feel authors should take a call on changing the title of the article to suit their study results.

  We are in concurrence with the Reviewer’s reasoning on the need for Title change. We propose to change the Title,  From “An evaluation of the interproximal contacts of the permanent dentition-A study cast based classification” To “Prevalence of different types of interproximal contacts in the permanent dentition – a study cast evaluation.”  

1. Inclusion and exclusion criteria were not set properly, as following major criteria were not taken into consideration;

1. Severity of the malocclusion determines the interproximal contact area type, authors failed to set the selection criteria for this.

We are grateful to the reviewer for pointing out this important parameter. While we do agree that malocclusion type would have an impact on the type of contacts, we did not consider the malocclusion severity/classification in the eligibility criteria as we had planned a separate study on the prevalence of the various types of interproximal contacts and different malocclusions. Kindly refer to the answer for Question No. 3a

1. A filled or carious or traumatic tooth would obliviously affect the interproximal contact point, authors fail to consider this as well.

We acknowledge and are grateful to the Reviewer for the observation. Though filled, carious and traumatic teeth were not mentioned as part of the exclusion criteria, they were excluded from the study. We have now added them in the exclusion criteria. Changes made in Materials and Methods From The exclusion criteria were any occlusal wear and any developmental anomalies affecting the morphology of the tooth (peg laterals, etc.) To The exclusion criteria were casts of patients with carious, filled or fractured teeth, occlusal wear and  any developmental anomalies affecting the morphology of the tooth (peg laterals, etc.)

1. Was there in criteria set to exclude periodontally compromised patients? would this have any affect on your findings.         

We concede that a compromised periodontium might affect the interproximal contatcts. With the study casts, a periodontal evaluation was difficult. Hence the periodontal status was not considered.   

1. Will the shape of tooth determine the types of interproximal contact area, was there any consideration given to this factor?

We endorse the Reviewer’s question. Yes, the shape of the tooth would affect the contact. However, teeth with anomalies affecting their morphology were part of the exclusion criteria. The exclusion criteria were the presence of caries, any occlusal wear and any developmental anomalies affecting the morphology of the tooth (peg laterals, etc.).  

1. Authors finding suggests that, the prevalence for 'x' type contact was more in midline teeth, lateral incisors and central incisors, but article by Stappert et al., suggests that "Contact areas, not contact points, were observed between neighboring maxillary anterior" teeth. (Stappert, Christian & Tarnow, Dennis & Tan-Chu, Jocelyn & Chu, Stephen. (2010). Proximal Contact Areas of the Maxillary Anterior Dentition. The International journal of periodontics & restorative dentistry. 30. 471-7.). Authors can give their feedback on the same          

We are grateful to the Reviewer for bringing this important study to our attention. The findings of Stappert et al have been added in the “Discussion” section of our manuscript. Changes made to Discussion – (Paragraph added and References changed) Stappert et al ^19^ reported that the quantum of the contact area decreased progressively from 4mm at the midline contact to 1.5mm at the canine premolar contact. They reported that contact areas, not contact points, were observed between neighbouring maxillary anterior teeth. The findings of our study were not in consonance with Stappert et al. 19 ^^While our study had a greater prevalence of “X” contacts, and therefore point contacts, the reason for this variation could be because Stappert et al ^19^ assessed the interproximal contacts on patients who had no crowding or spacing in the anterior teeth. Thus, it would not be representative of our sample which had malocclusion and therefore malaligned teeth. Further, our study was a linear measurement based on visual observation of the interproximal contact while Stappert et al ^20^ quantified the area based on mathematical calculations.
